# Emergence of Oxacillin Resistance in Stealth Methicillin-Resistant *Staphylococcus aureus* Due to *mecA* Sequence Instability

**DOI:** 10.1128/AAC.00558-19

**Published:** 2019-07-25

**Authors:** Richard V. Goering, Erin A. Swartzendruber, Anne E. Obradovich, Isabella A. Tickler, Fred C. Tenover

**Affiliations:** a Department of Medical Microbiology and Immunology, Creighton University School of Medicine, Omaha, Nebraska, USA; b Cepheid, Sunnyvale, California, USA

**Keywords:** MRSA, emergence of resistance, *mecA*, oxacillin susceptible, whole-genome sequencing

## Abstract

Staphylococcus aureus strains that possess a *mecA* gene but are phenotypically susceptible to oxacillin and cefoxitin (OS-MRSA) have been recognized for over a decade and are a challenge for diagnostic laboratories. The mechanisms underlying the discrepancy vary from isolate to isolate. We characterized seven OS-MRSA clinical isolates of six different *spa* types from six different states by whole-genome sequencing to identify the nucleotide sequence changes leading to the OS-MRSA phenotype. The results demonstrated that oxacillin susceptibility was associated with mutations in regions of nucleotide repeats within *mecA*. Subinhibitory antibiotic exposure selected for secondary *mecA* mutations that restored oxacillin resistance. Thus, strains of S. aureus that contain *mecA* but are phenotypically susceptible can become resistant after antibiotic exposure, which may result in treatment failure. OS-MRSA warrant follow-up susceptibility testing to ensure detection of resistant revertants.

## Abstract (precis)

Staphylococcus aureus strains that possess a *mecA* gene but are phenotypically susceptible to oxacillin and cefoxitin (OS-MRSA) have been recognized for over a decade and are a challenge for diagnostic laboratories. The mechanisms underlying the discrepancy vary from isolate to isolate.

## INTRODUCTION

Infections caused by methicillin-resistant Staphylococcus aureus (MRSA) are an issue of long-standing global concern (1). As with many problem pathogens, efforts to improve both detection and therapeutic outcome now commonly rely on a combination of traditional and molecular approaches (e.g., disk diffusion and PCR, respectively). However, instances may arise where phenotypic and genotypic data appear discrepant. Such is the case with Staphylococcus aureus isolates appearing susceptible to oxacillin or cefoxitin by disk diffusion or MIC testing but positive for the *mecA* gene by PCR, commonly termed oxacillin-susceptible MRSA (OS-MRSA) (2, 3). For over a decade clinical OS-MRSA isolates have been reported from various geographic locations, including continental Europe (3–5), the United Kingdom (6), North America (7–9), Asia (10–15), Africa (16), and South America (17, 18). A similar phenomenon has also been seen in livestock-associated and environmental isolates (10, 19, 20). Each report has underscored the diagnostic dilemma such strains represent. However, most studies have not addressed the potential cause(s) of phenotypic-genotypic disparity. Investigations that have examined underlying mechanisms have generally found heterogeneous bacterial populations with either (i) a resistant subset or (ii) induction of resistance among susceptible cells (7, 16, 21). In the present study, a group of North American S. aureus clinical isolates with a clear propensity to produce resistant colonies within the zone of inhibition in cefoxitin disk susceptibility tests were analyzed by whole-genome sequencing (WGS) to investigate the nature of the mechanism(s) underlying the OS-MRSA phenotype.

## RESULTS

**FIG 1:**
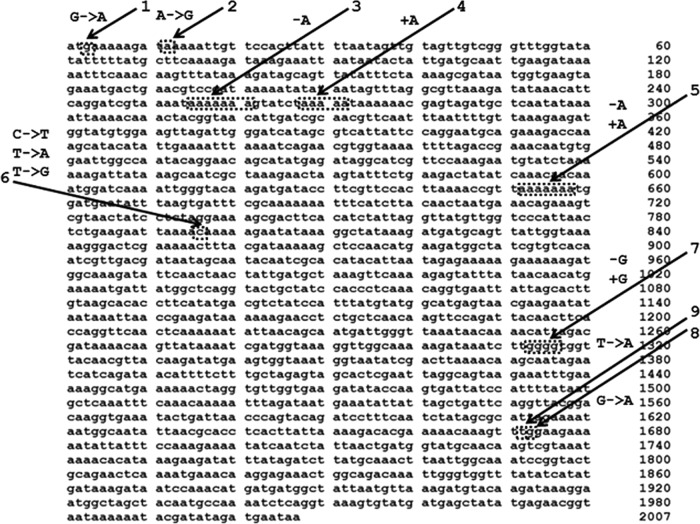
Comparison of *mecA* sequences in OS-MRSA and MRSA revertants using the wild-type *mecA* sequence from S. aureus strain MW2 (CP026073.1) as a template. The location of specific sequence changes are numerically referenced and referred to in Table 2.

The seven OS-MRSA study isolates from six U.S. states represented six *spa* types, three multilocus sequence types (MLST), and two SCC*mec* types (Table 1). Prior to antibiotic exposure, the isolates were determined to be cefoxitin screen negative and oxacillin susceptible by MIC testing, cefoxitin susceptible by disk diffusion testing, PBP2a negative by latex agglutination (isolate CRG2943 was weakly positive), and *mecA* PCR positive. While detailed clinical information was not available, six of the isolates were cultured from active infections two of which (CRG2382 and CRG2383) were from the same hospital but epidemiologically unrelated patients and were previously reported to appear to show “inducible” resistance (7). One isolate (CRG2935) was from a nasal surveillance culture. The four variations of the disk susceptibility testing method were associated with minor differences in the appearance of cefoxitin-resistant isolates. For example, resistant colonies were always more easily detected (i.e., larger colonies appearing earlier during incubation) on brain heart infusion (BHI) medium with a 10^7^ CFU/ml inoculum. This was especially true for CRG2937, CRG2939, and CRG2941, which produced pinpoint colonies within the zones of inhibition at 24 h. After 48 h of incubation, all of the isolates produced colonies within the zones of inhibition of the disks. However, for CRG2382, CRG2383, CRG2935, and CRG2943 the colonies were typically near or at the edge of the zone of inhibition, with standard susceptibility testing using Mueller-Hinton agar (MHA, 10^5^ CFU/ml) giving smaller and less frequently seen numbers of colonies. These colonies were subsequently shown to be resistant by MIC and disk diffusion testing (Table 1). Similar results were observed using either oxacillin or cefazolin disks. To investigate the genetic basis for the resistant colonies detected by disk diffusion, the cefoxitin-susceptible parent strains and their resistant derivatives were analyzed by WGS. Sequence analysis of the *mecA* gene in the isolates (referenced against S. aureus MW2; accession number CP026073.1) (Fig. 1) revealed 21 instances of five or more T or A tandem repeats, with additional longer sequences interrupted by only a single alternative base. The seven OS-MRSA isolates exhibited a variety of single point or reading frame mutations (i.e., stop codons) usually associated with tandem repeat sequences (Fig. 1; Table 2) likely due to slip-strand mispairing during DNA replication (22). In each case, exposure to subinhibitory concentrations of antibiotic selected for a corrective secondary mutation (Fig. 1; Table 2), which restored *mecA* function (i.e., PBP2a production) and cefoxitin resistance at frequencies ranging from ∼1 × 10^6^ to 1 × 10^7^, which was stable even after five subcultures on drug-free BHI agar. Further WGS data analysis revealed no obvious defects in genes associated with DNA replication, repair, or mutator function which might predispose to the OS-MRSA phenotype. This was also confirmed by assessing the rates of spontaneous mutation to rifampin resistance for each of the isolates (23), which did not differ significantly from conventional MRSA isolates (data not shown), supporting the absence of a mutator genotype.

**TABLE 1:** Characteristics of oxacillin-susceptible MRSA clinical isolates and their MRSA revertants^*a*^

Isolate	Origin (U.S. state)	Source	*spa* type	SCC*mec* type	MLST ST/CC	Before exposure to antibiotic			After exposure to antibiotic		
						Oxacillin MIC (μg/ml)	Cefoxitin screen (μg/ml)	PBP2a	Oxacillin MIC (μg/ml)	Cefoxitin screen (μg/ml)	PBP2a
CRG2382	KS	SSTI^*b*^	t175	IV	ST1/CC1	0.5	≤4	Neg	>2	>4	Pos
CRG2383	KS	SSTI	t175	IV	ST1/CC1	0.5	≤4	Neg	>2	>4	Pos
CRG2935	IA	Nares	t002	IV	ST5/CC5	≤0.25	≤4	Neg	>2	>4	Pos
CRG2937	OR	Blood	t242	II	ST5/CC5	≤0.25	≤4	Neg	>2	>4	Pos
CRG2939	NC	Blood	t2308	II	ST105/CC5	≤0.25	≤4	Neg	1	>4	Pos
CRG2941	TN	Blood	t010	II	ST5/CC5	≤0.25	≤4	Neg	>2	>4	Pos
CRG2943	MS	Blood	t002	IV	ST5/CC5	≤0.25	≤4	Weak Pos	>2	>4	Pos

a MRSA clinical isolates were *mecA* positive but susceptible to cefoxitin by disk diffusion (zone size, ≥22 mm). MRSA revertants were resistant to cefoxitin by disk diffusion (zone size, ≤21 mm). Pos, positive; Neg, negative.

b SSTI, skin and soft tissue infection.

**TABLE 2:** Comparison of *mecA* sequences in OS-MRSA strains and MRSA revertants^*a*^

Isolate^*b*^	OS-MRSA		MRSA revertant		
	Relevant *mecA* sequence	Result	Relevant *mecA* sequence	Result	Position(s) on *mecA* sequence map
CRG2382	C→T, nt 796	Stop codon replaces glutamine	T→A, nt 796	Lysine replaces stop codon	6
CRG2383	C→T, nt 796	Stop codon replaces glutamine	T→G, nt 796	Glutamic acid replaces stop codon	6
CRG2935	G→A, nt 1673	Stop codon replaces tryptophan	T→A, nt 1672	Lysine replaces stop codon	8, 9
CRG2937	G→A, nt 3	Stop codon replaces methionine	A→G, nt 12	New methionine created (only first three aa lost)	1, 2
CRG2939	Loss of A, nt 255–261	Reading frameshift produces stop codon	Insertion of A, nt 268–272	Reading frame restored	3, 4
CRG2941	Loss of A, nt 662–668	Reading frameshift produces stop codon	Insertion of A, nt 662–668	Reading frame restored	5
CRG2943	Loss of G, nt 692–695	Reading frameshift produces stop codon	Insertion of G, nt 692–695	Reading frame restored	7

a “Relevant *mecA* sequence” columns indicate the nucleotide change and the change site(s). nt, nucleotide(s); aa, amino acid(s).

b Isolates CRG2382 and CRG2383 were obtained from different patients during different time periods (7).

## DISCUSSION

The diagnostic dilemma posed by OS-MRSA was recently summarized by Tenover and Tickler in a literature survey documenting the presence of such strains in 1 to 25% of the isolates examined (2). While these survey numbers represent OS-MRSA arising by a variety of mechanisms and different susceptibility testing methods, the most problematic are the subset of PBP2a-negative isolates, such as those seen here, which represent true “stealth” MRSA with a high potential for reversion to resistance in the presence of antibiotic. A previous study by Proulx et al. (24) reported an OS-MRSA isolate with a *mecA* frameshift mutation similar to that seen with isolate CRG2939 reported here. However, our study extends understanding of this issue by demonstrating that such OS-MRSA may be found in a variety of strain backgrounds (e.g., multiple *spa* and MLST types), clinical specimens (blood and wounds), and geographic locations (multiple states in the United States). Events contributing to the initial *mecA* mutations in these OS-MRSA strains leading to phenotypic susceptibility are unknown. However, the “fluid” nature of OS-MRSA reversion is clearly evidenced by isolates such as CRG2382 and CRG2383. Both were from the same health care facility in Kansas but were from different patients and obtained during different time periods. Both isolates had identical *mecA* mutations initially, but they were reversed by different secondary mutations. As noted above and in contrast to Proulx et al. (24), antibiotic exposure at subinhibitory concentrations did select for reversion to resistance. However, previous population analysis of these two isolates (7) demonstrated their pure “MSSA-like” nature, confirming that such strains represent a stable homogeneous genetic background, appearing antibiotic susceptible but with a clear (but masked) potential for emergence of resistance under appropriate conditions. This includes subinhibitory antibiotic concentrations, which can definitely occur in a clinical environment. The “stealth” nature of these MRSA underscores the importance of vigilance with regard to their accurate detection. DNA sequencing is required to reveal the presence of *mecA* mutations, such as those seen here, that render MRSA phenotypically susceptible but fully capable of reversion to resistance when exposed to an antimicrobial agent. However, all OS-MRSA do not have the same genetic basis. Thus, the extent of the sequencing needed to identify mutations responsible for the OS-MRSA phenotype would be broad, which is currently not practical for laboratories performing MIC tests. Tenover and Tickler (2) have recently summarized useful approaches to the general detection of OS-MRSA. For isolates such as those described here, colonies within the zone of inhibition in disk susceptibility tests (especially with enriched medium and extended incubation) are a clue arguing in favor of a provisional report of MRSA since the potential exists for relatively rapid and stable emergence of resistance. While approaches to accurate pathogen diagnosis, characterization, and treatment continue to advance, these data underscore the complex interplay between phenotype and genotype in MRSA strains.

In summary, we have demonstrated that the inherent instability of tandem base repeats within the S. aureus *mecA* sequence can produce “stealth” MRSA (OS-MRSA) in different strain backgrounds and clinical settings. These strains are capable of reversion to resistance by simple and relatively frequent point mutation to restore gene function, underscoring the importance of vigilance in the diagnosis and treatment of these problem pathogens.

## MATERIALS AND METHODS

### Bacterial isolates.

The characteristics of the clinical isolates examined are summarized in Table 1. Determination of *spa* type and SCC*mec* type were performed by published methods (25). PBP2a production was assessed using commercially available products (Oxoid PBP2′ latex agglutination test [Oxoid Microbiology Products, Basingstoke, Hampshire, United Kingdom] and Alere PBP2a [Alere, Waltham, MA]). Antimicrobial susceptibility testing was performed using the MicroScan Walkaway Pos MIC Panels type 29 (Beckman Coulter, Brea, CA) according to the manufacturer’s instructions. The isolates were also tested using the disk diffusion method, according to Clinical and Laboratory Standards Institute (CLSI) guidelines (26) using cefoxitin disks and interpreted using CLSI M100 A-28 (27). Quality control organisms for antimicrobial susceptibility testing included Staphylococcus aureus ATCC 29213, S. aureus ATCC 25923, and S. aureus ATCC BAA-977 (all methicillin susceptible), as well as ATCC 43300 (MRSA), Enterococcus faecalis ATCC 29212, and Escherichia coli ATCC 35218.

### Conventional and whole-genome sequencing.

“Sanger” *mecA* sequence analysis of isolates was performed using previously described primers (28). For WGS, genomic DNA was extracted (DNeasy kit; Qiagen, Germantown, MD), and Nextera XT libraries were sequenced on an MiSeq instrument according to the manufacturer’s instructions (Illumina, San Diego, CA). WGS analysis was performed using BioNumerics (v.7.6; Applied Maths, Belgium).

### Mutation to antibiotic resistance.

To define conditions influencing the production of resistant colonies by the isolates, four variations of cefoxitin disk susceptibility testing were investigated. We used both standard (10^5^ CFU/ml) and elevated (10^7^ CFU/ml) inocula on MHA (BD Difco, Sparks, MD) and BHI agar (BD Difco) plates that were incubated at 35°C and examined at 24 and 48 h. To determine the frequency of cefoxitin-resistant derivatives of the OS-MRSA isolates, ∼10^7^ cells were inoculated onto BHI agar plates (BD Difco) containing cefoxitin (4 μg/ml), which were subsequently replica plated to cefoxitin-containing agar plates (8 μg/ml) to confirm resistance. The propensity for an isolate to mutate (i.e., mutator genotype) was assessed by determining the isolate’s spontaneous mutation frequency to rifampin resistance as previously described (23).
